# Colonic interposition between the liver and left diaphragm - management of Chilaiditi syndrome: A case report and literature review

**DOI:** 10.3892/ol.2014.1903

**Published:** 2014-02-21

**Authors:** WEI-HONG WENG, DA-REN LIU, CHENG-CHENG FENG, RI-SHENG QUE

**Affiliations:** 1 Department of General Surgery, The Second Affiliated Hospital, Zhejiang University, Hangzhou, Zhejiang 310009, P.R. China; 2 Department of Surgery, Cixi Red Cross Hospital, Cixi, Zhejiang 315300, P.R. China

**Keywords:** hepatodiaphragmatic interposition, Chilaiditi sign, Chilaiditi syndrome

## Abstract

Chilaiditi syndrome refers to a medical condition that is indicated by the presence of Chilaiditi sign, the radiological observation of a colonic interposition between the liver and the diaphragm, and is associated with other clinical symptoms. Chilaiditi syndrome is a rare entity and therefore, is often misdiagnosed in clinical practice, however, it may be accompanied by a series of severe complications, such as bowel obstruction and perforation. The current study describes a 47-year-old male who presented with repeated abdominal pain and acute intestinal obstruction. The patient was diagnosed with Chilaiditi syndrome via radiological observation and was cured by conservative treatment. The clinical data of seven additional patients with Chilaiditi syndrome, which was reported in the Chinese literature between January 1990 and January 2013, were also collected. The pathogenesis, clinical manifestation, diagnosis and treatment of this syndrome have been reviewed and analyzed. The current study may be useful to familiarize clinical practitioners with Chilaiditi syndrome, in order to avoid a misdiagnosis during clinical treatment.

## Introduction

Chilaiditi sign refers to the radiological observation of segmental interposition of the colon between the liver and the diaphragm, which was first described by Chilaiditi, a Greek radiologist, in 1910 (1). Chilaiditi sign has an incidence of 0.025–0.28% worldwide (2) with a marked male predominance (male to female, 4:1) (3). Chilaiditi sign may be asymptomatic or may be accompanied by a series of clinical symptoms, including digestive symptoms, which range from mild abdominal pain to acute bowel obstruction and is termed Chilaiditi syndrome (4). The management of Chilaiditi syndrome includes conservative treatment and surgical intervention. Chilaiditi syndrome is often misdiagnosed in clinical practice due to its rarity, particularly in China. The current study presents a patient with Chilaiditi syndrome who was treated in January 2012, in addition to a review of seven cases of Chilaiditi syndrome obtained from the Chinese literature between 1990 and 2013. The pathogenesis, clinical manifestation, diagnosis and treatment of Chilaiditi syndrome are analyzed and discussed. The patient provided written informed consent.

## Case report

### Case report

**Figure 1:**
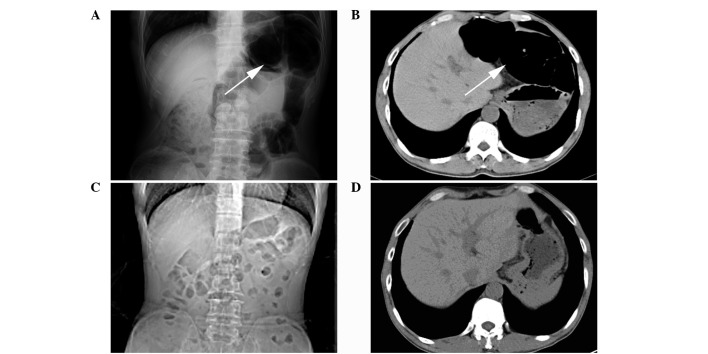
Radiological observations. (A) Plain abdominal X-ray revealed a gas shadow with visible haustral folds in the left subphrenic space (indicated by the arrow) prior to treatment. (B) Computed tomography (CT) identified an interposition of the left colon between the liver and the left diaphragm (indicated by the arrow) prior to treatment. (C and D) The gas shadow in the left subphrenic space and the colonic interposition in the CT scan disappeared following conservative treatment for one week.

A 47-year-old male was admitted to the Emergency Department of the Second Affiliated Hospital, Zhejiang University (Hangzhou, China) in January 2012, presenting >40 years of intermittent upper abdominal pain and the absence of flatus for six days. The symptoms were associated with abdominal distension, nausea and vomiting, which were relieved by a change of posture or a sudden movement. The patient had no history of surgery. The physical examination revealed a soft abdomen with mild upper abdominal tenderness; no rebound tenderness or muscle guarding was identified. In addition, auscultation revealed hypoactive bowel sounds. A plain abdominal X-ray showed an abnormal gas shadow in the left subphrenic space and a segment of gaseous distended colon, which was located in the left side of the abdominal cavity; this was interposed between the liver and the left diaphragm, which indicated a large bowel obstruction (Fig. 1A). Computed tomography (CT) confirmed the interposition of the colon (Fig. 1B) accompanied by the small bowel distention with several air-fluid levels. The patient was treated conservatively with fasting, nasogastric decompression, fluid supplementation, parenteral nutrition, enemas and the application of somatostatin. Six days later, the patient was able to pass stools and tolerate a liquid diet. After one week, CT was performed again, which showed normal organ morphology in the left upper quadrant and identified that the distended colon between the liver and the diaphragm was restored to its normal position. In addition, the air-fluid levels in the small bowel had also disappeared (Fig. 1C and D). The patient received no further surgical treatment and no evidence of recurrence was found during the one year of follow-up.

### Literature review

The Chinese Biology and Medicine Database (http://sinomed.imicams.ac.cn/zh/), the Chinese Periodical Database of Science and Technology (http://lib.cqvip.com/), and the China Hospital Knowledge Database (http://www.chkd.cnki.net) were searched for cases of Chilaiditi syndrome between January 1990 and January 2013. Duplicate reports were excluded to avoid over-representation and seven cases of Chilaiditi syndrome, as reported in the Chinese literature (5–9) were identified (Table I).

The median age of the seven patients at presentation was 60 years (range, 39–75 years) and the male to female ratio was 4:3. The shortest duration of the symptoms was ~5 h of acute bowel obstruction in a 39-year-old male, and the longest was >40 years in a patient who presented with repeated abdominal pain and constipation. The most frequent symptom of Chilaiditi syndrome was abdominal pain (n=6/7) with abdominal tenderness (n=3/7). All of the cases were diagnosed by CT, in which the distension of the colon interposed between the liver and the diaphragm was revealed. One patient exhibited atrophy of the right lobe of the liver and an additional patient exhibited the anatomic abnormality of a congenital liver split. In addition, a plain abdominal or chest X-ray was performed (n=4/7), which showed a bowel shadow in the subphrenic space. The treatment included conservative treatment (n=4/7) and surgical intervention (n=3/7). The conservative treatment included fasting, nasogastric decompression, fluid supplementation, enemas, peristalsis stimulation, traditional Chinese medicine (auricular acupressure of *Vaccaria*) and acupuncture. One patient underwent a right hemicolectomy and during surgery, an exceptionally elongated and distended right colon was revealed. Furthermore, a dense adhesion was identified between the colon and the lower edge of the right hepatic lobe as well as atrophy of the right hepatic lobe. Hu (6) reported two cases of Chilaiditi syndrome that were identified with malignancies (rectal cancer and mesenteric lymphosarcoma) during an exploratory laparotomy, in which extensive peritoneal metastasis, adhesions, laxity of the right triangular ligament, and the insertion of a distended transverse colon between the liver and the diaphragm were revealed. The seven patients recovered well with the exception of the cases that were accompanied by a malignancy. The follow-up data were only available for one case that demonstrated no evidence of recurrence for one month.

## Discussion

Intestinal interposition is a medical condition where a segment of the bowel is temporarily or permanently interposed between two organs, for example the liver and the diaphragm, the spleen and the diaphragm, the spleen and the left kidney or the stomach and the pancreas. Among these, the hepatodiaphragmatic interposition is termed Chilaiditi sign and the others are termed non-Chilaiditi sign (10). The interposed bowel is usually the hepatic flexure of the colon and much less frequently the small bowels, which occurs in 3–5% of Chilaiditi sign cases (11). Chilaiditi sign is usually revealed incidentally by chest or abdominal radiographs, with an incidence of 0.025–0.28%. In addition, Chilaiditi sign is usually asymptomatic and when accompanied with clinical symptoms is termed Chilaiditi syndrome. The incidence of Chilaiditi syndrome rises with increasing age (12) and has a marked male predominance (male to female ratio, 4:1) (3).

Normally, suspensory ligaments and fixation of the colon impede the interposition of the colon between the liver and the diaphragm. However, various factors have been implicated that result in the pathological interposition of the colon, these include hepatic, intestinal, diaphragmatic and other miscellaneous causes. A large space between the liver and the diaphragm may potentially lead to colonic interposition (13). Additional factors include those relating to the intestines, such as megacolon, an elongated/hypermobile colon with constipation (5), absence/laxity/elongation of the ligament suspending the transverse colon, abnormal gas accumulation due to aerophagia. The diaphragmatic factors include the rise of the right hemidiaphragm, such as eventration or phrenic nerve injury, and the hepatic factors include atrophy of the liver due to cirrhosis or congenital etiology (for example a congenital liver split or relaxation/elongation of suspensory ligaments). Other factors include enlargement of the lower thoracic cavity (chronic obstructive pulmonary disease), increased intra-abdominal pressure (obesity, multiple pregnancies and ascites), mental retardation and schizophrenia, which are also associated with anatomic abnormalities that result in Chilaiditi sign (14). Intraperitoneal adhesion, which is caused by widespread tumor metastasis or previous surgery is also one of the factors (6). In addition, psychotropic medication (15) and iatrogenic factors (16), such as endoscopic procedures, have been reported as causative factors. In the present case, the patient exhibited a childhood-onset symptom, therefore, interposition of the colon was more likely due to a congenital anatomical anomaly. In addition, the patient had emphysema and chronic obstructive pulmonary disease, therefore, enlargement of the lower thoracic cavity may also have been a possible cause.

Chilaiditi syndrome has various clinical manifestations, such as decreased appetite, abdominal pain, flatulence, nausea, vomiting and constipation. The seven cases reviewed in the current study all presented with gastrointestinal symptoms with the exception of one patient. However, in rare cases, other symptoms besides those associated with the gastrointestinal tract, such as respiratory distress and angina-like chest pain, may also be observed (17) Thus, Chilaiditi syndrome must be considered in chest pain patients with electrocardiogram results, cardiac function and cardiac enzymes within the normal ranges. In addition, Chilaiditi syndrome may also be an indirect manifestation of certain abdominal malignancies, with or without peritoneal metastasis. In the present study, two of the previously reported cases were accompanied with malignancies, one with rectal cancer and the other with mesenteric lymphosarcoma (6). The complications of Chilaiditi syndrome may include intestinal obstruction, volvulus and perforation. In rare cases perforated subdiaphragmatic appendicitis may occur as a complication of Chilaiditi syndrome (18).

A characteristic marker of Chilaiditi sign is the observation of air below the diaphragm, with visible haustral folds or valvulae conniventes between the liver and the diaphragmatic surface. In addition, the location of the air is not changed by altering the posture of the patient. Chilaiditi sign must be differentiated from pneumoperitoneum by X-ray. Pneumoperitoneum normally shows a crescent-shaped gas shadow under the diaphragm without haustral folds or valvulae conniventes, and altering the posture of the patient changes the position of the gas. Patients with pneumoperitoneum always exhibit injury to the hollow viscus and simultaneously possess signs of peritonitis. Ultrasonography is also useful in the differentiation of Chilaiditi syndrome from pneumoperitoneum, which usually requires an immediate surgical intervention (19).

It is important to identify colonic interposition in patients that are predisposed to developing Chilaiditi sign, such as cirrhotic patients, in order to prevent complications during a percutaneous transhepatic procedure or liver biopsy (14). In addition, colonoscopies must be performed carefully in patients with Chilaiditi sign to prevent intestinal perforation.

Interventions are not required for asymptomatic patients with Chilaiditi sign and the treatment is usually conservative. The present case and four of the reviewed cases were all cured by conservative treatment, which included bed rest, nasogastric decompression, fluid supplementation, enema, laxatives and the discontinuation of offending medications (for example psychotropic medications) (20). In addition, traditional Chinese medicine may be used as an alternative; the two cases reported by Su *et al* (8) were cured by acupuncture and auricular acupressure of *Vaccaria*. When conservative treatment fails, the intestinal obstruction, such as volvulus, intestinal ischemia or intestinal perforation, can not be alleviated and thus, surgical intervention is required. Saber and Boros (21) previously reported that 26% of patients require operative management and that the number of surgical interventions for long-term intermittent abdominal pain continues to increase. Surgical interventions include segmental colon resection, colopexy and hepatopexy (15).

In conclusion, Chilaiditi syndrome is rare in China and may be asymptomatic or present with acute abdominal pain. Occasionally, Chilaiditi syndrome is associated with malignancies and may, therefore, be misdiagnosed. The present study allows clinicians to become familiarized with this syndrome and its management, in order to avoid a misdiagnosis during clinical treatment.

## Figures and Tables

**Table I:** Clinical data of seven cases.

First author (year) [ref]	Age (years)	Gender	Clinical manifestation	Auxiliary examination	Complication	Treatment
Wang *et al* (2012) [5]	48	Female	Abdo pain and constipation	Chest X-ray and CT	Atrophy of right liver lobe	Right hemicolectomy
Hu (2007) [6]	70	Male	Abdo pain and bloody stools	Abdo X-ray and CT	Rectal cancer	Exploratory laparotomy
Hu (2007) [6]	60	Male	Mass in left lower quadrant	Abdo CT	Mesenteric lymphosarcoma	Exploratory laparotomy
Cui *et al* (2012) [7]	65	Male	Abdo pain	Abdo CT	None	Conservative
Su *et al* (2005) [8]	62	Female	Abdo pain and constipation	Abdo X-ray and CT	None	Conservative
Su *et al* (2005) [8]	75	Female	Abdo pain and constipation	Chest and abdo X-ray and CT	None	Conservative
Shen (2000) [9]	39	Male	Abdo pain	Abdo CT	Congenital liver split	Conservative

Abdo, abdominal; CT, computed tomography.
